# Caregiver perspectives on TB-related stigma experienced by young children

**DOI:** 10.5588/ijtldopen.25.0293

**Published:** 2025-08-13

**Authors:** L.S. Johnson, M.G. Anthony, C. Purdy, V. Luke, H. van Deventer, M. van Niekerk, L. Viljoen, M.M. van der Zalm

**Affiliations:** ^1^ Desmond Tutu TB Centre, Stellenbosch University, South Africa;; ^2^ Program for Public Health, Northwestern University, USA.

**Keywords:** tuberculosis, paediatric TB, South Africa, psychosocial well-being

## Abstract

**BACKGROUND:**

TB-related stigma often stems from a fear of TB infection, power dynamics between social groups, and an association of TB with socially undesirable traits.

**METHODS:**

This study was conducted in South Africa within a prospective observational TB diagnostic cohort study, ‘Umoya.’ StopTB stigma questionnaires and activity-based interviews were administered to caregivers of children aged 0–9 years with presumptive pulmonary TB (PTB) 16 to 24 weeks after enrollment.

**RESULTS:**

In total, 64 caregivers of 70 children (median age: 2y) with PTB completed the questionnaire. Most children (56%) had a known TB contact in the household. The questionnaire revealed that anticipated stigma was a common concern, with worries about people gossiping or speaking badly about their children (16.7%) or their child’s feelings being hurt because of their TB diagnosis (16.7%). Internalized stigma of the child, as perceived by their caregiver, was the least affirmed stigma domain. Overall, caregiver perceptions of internalized stigma did not delay treatment. Twelve of these caregivers were also interviewed, which demonstrated themes of anticipated and internalized stigma, and comparisons to HIV stigma.

**CONCLUSION:**

Deepening our understanding of stigma is critical to improving outcomes and experiences of young children and their families affected by TB.

TB remains an ongoing global health threat. Children under 15 years accounted for an estimated 12% of all TB worldwide in 2023, resulting in 1.3 million children with TB disease and over 180,000 deaths.^1^ Although children are less likely to be infectious, they are more likely to progress from TB infection to disease.^2^ In countries with a high TB burden, including South Africa, TB remains a significant cause of mortality and morbidity, particularly among children under five years of age.^3^ Stigma, the process through which a characteristic or condition is deemed undesirable, significantly contributes to the ongoing spread of TB.^4^ Stigma can impede TB diagnosis, healthcare utilization, treatment acceptance and adherence, and overall psychosocial well-being.^5–8^ TB stigma commonly stems from a fear of infection, lack of TB knowledge, and associations with socially undesirable conditions, such as poverty.^5,9,10^ Stigma can appear in various forms: gossip, isolation, or mistreatment due to association with a stigmatized group (enacted or secondary due to association), fear or expectation of mistreatment (anticipated), and the adoption of negative beliefs about oneself or self-isolation (internalized).^5,11,12^

Stigma can have profound consequences for children and adolescents, including isolation from friends and the broader community, poor mental health, and disrupted education.^13,14^ TB stigma has been observed in South African settings among adolescents, children, and their caregivers.^15,16^ However, to our knowledge, no studies have specifically sought to understand how caregivers of young children with TB perceive stigma in this setting. This study aimed to explore caregivers' experiences and perceptions of TB-related stigma among young children (0–13 years) diagnosed with pulmonary TB (PTB) in Cape Town, South Africa. Using a mixed-methods approach with a novel discussion guide, we sought to offer unique insights into how TB stigma is experienced across various socio-ecological levels, including the household and community. Additionally, we examined how TB and associated stigma affect both caregivers’ and their children's psychosocial well-being and health-seeking behaviors.

## METHODS

### Study design, population

This cross-sectional study was conducted within a broader prospective longitudinal TB diagnostic cohort study, ‘Umoya,’ which aims to improve TB diagnosis and assess the long-term impacts of PTB on children in Cape Town, South Africa.^17^ The study includes children aged 0–13 years, of whom ±80% are between the ages of 0–5. This age distribution reflects the high prevalence of TB among children under 5, compared to children 5–14.^18^ Children with presumptive PTB were recruited from two secondary and tertiary level public hospitals, Karl Bremer and Tygerberg, and surrounding primary care clinics in the Cape Metropolitan District (CMD). Socio-demographic and clinical information was collected at the participants’ baseline visit (n=600). Most caregivers with children with microbiologically confirmed and clinically diagnosed TB completed an adapted Stop TB questionnaire^19^ at their week 16 visit. Data were collected from March 2021 to March 2024. A subset of participants and their caregivers were invited for an interview by the Umoya study coordinator at either week 16 or week 24, depending on the caregiver’s willingness and availability to participate after their child completed all other study assessments. Participants who were unable to stay at the study clinic were offered transportation assistance to return for an interview within two weeks.

### Setting

This study was implemented in Cape Town, Western Cape Province, South Africa. The CMD healthcare system faces challenges due to an ongoing high burden of disease, infrastructural issues, and social inequities.^20^ Umoya participants are commonly recruited from the informal settlements of Delft, Wallacedene, Bloekombos, and Khayelitsha, areas with high poverty rates and significant disparities in healthcare access.^21^

### Data collection

Supervised by socio-behavioral scientists, the study counselor administered the questionnaire^19^ (see Supplementary Data) in the home language of participants (English, Xhosa, or Afrikaans) and entered responses into the Research Electronic Data Capture (REDCap) platform.^22^ Individuals who brought the child to the study visit and identified as the primary caretaker of the child at baseline, or at week 16 or week 24 interviews, were considered caregivers. Caregivers were asked about their level of agreement with 23 statements. Post-graduate research assistants fluent in the participants' preferred language (as above) conducted one-on-one interviews using a semi-structured interview guide developed by the research team (see Supplementary Data). The guide was informed by the Health Stigma and Discrimination Framework (HSDF)^12^ and divided into three sections: (1) perceptions of stigma, where participants were asked to look at ‘character cards’ (see Supplementary Data) to discuss hypothetical people with a TB diagnosis; (2) personal stigma experiences and (3) responses to definitions of each stigma domain. Based on initial interview responses, the guide was iteratively refined with input from senior researchers (LV, MMVDZ). Each interview was audio-recorded and lasted 30–60 minutes. Following interviews, research assistants wrote reflective case descriptions^23^ and included verbatim translated quotes illustrating the participants' perspectives.

### Analysis

The adapted Stop TB questionnaire items were categorized into stigma domains according to the HSDF.^12^ Questionnaire responses of ‘Strongly Agree’ and ‘Agree’ and ‘Neutral’, ‘Disagree’, and ‘Strongly Disagree’ were combined. Descriptive summary statistics were produced using R^24^ to characterize the children and their caregivers.^12^ Case descriptions of interviews were thematically analyzed^25^ for comparison. The first author (LSJ) and two additional research assistants (CP and VL) coded each transcript twice. Reflective discussions were held to reach a consensus.

### Ethics

This study was approved by the Stellenbosch University Health Research Ethics Committee as part of the UMOYA study (N17/08/083). Prior to the questionnaire and interview, participants were informed that they could withdraw from the study at any time without forfeiting their participation in the overall clinical study. All caregivers provided written informed consent prior to participation.

## RESULTS

Caregivers completed the questionnaire for 70 children, which included three sibling pairs, representing 64 households (Table). In total, questionnaires were completed for 42 female children (60.0%) and 64 caregivers identified as the child's mother (90.9%). The median age of patients was 2 years [IQR: 1,3]. The median household size was 6 people [IQR: 5,8], with most households living in formal, brick housing (40/64; 62.5%). Over two-thirds of households reported receiving government grants (46/64; 73.0%). Participants spoke Afrikaans (31/64; 48.4%), Xhosa (22/64; 34.4%), and English (11/64; 17.2%). Forty children (57.1%) had a known TB contact, commonly identified as the primary caregiver (16/40; 40.0%) or another household member (24/40; 60.0%). Slightly less than one-third of children were HIV exposed (20/70; 29.0%). Twelve caregivers of 13 children with TB were interviewed (Table 1). Eight caregivers identified as the child's mother (66.7%). Other caregivers included a father (1/12; 8.3%), a grandparent (1/12; 8.3%), and other family members (2/12; 16.7%). The median age of the children was 3 [IQR:1,5]. The median household size was about 7 people [IQR: 5,8], with most households living in formal, brick housing (11/12; 91.7%). Most households reported receiving government subsidies (10/12; 83.3%). Caregivers spoke either Afrikaans (8/12; 66.7%) or Xhosa (4/12; 33.3%). Ten (76.9%) children had a known TB contact, commonly the primary caregiver (4/10; 40%) or another household member (4/10; 40%). Only two children had known exposure to HIV (2/13; 15.4%).

**Table.** Sociodemographic characteristics of participants.

Characteristics n (%) or median [IQR]	Questionnaires (n=70 children of 64 households)	Interviews (n=13 children of 12 households)
**Child gender**		
Female	42/70 (60.0)	8/13 (61.5)
**Child age, mean**	2 [1,3]	3 [1,5]
**Primary caregiver**	Reported at Baseline	Reported at Interview
Mother	58/64 (90.6)	8/12 (66.7)
Father	1/64 (1.6)	1/12 (8.3)
Grandparent	2/64 (3.1)	1/12 (8.3)
Other family member	3/64 (4.7)	2/12 (16.7)
**Household language preference**		
Afrikaans	31/64 (48.4)	8/12 (66.7)
English	11/64 (17.2)	0 (0.0)
Xhosa	22/64 (34.4)	4/12 (33.3)
**Household size**	6 [5,8]^B^	6.5 [5,8]
**Housing type**		
Formal (Brick)	40/64 (62.5)	11/12 (91.7)
Informal	12/64 (18.8)	0 (0.0)
Wendy house^A^	12/64 (18.8)	1/12 (8.3)
**Government grant recipient**		
Yes	46/64 (73.0)	10/12 (83.3)
No	17/64 (27.0)	2/12 (16.7)
**Someone employed in household**		
Yes	17/64 (26.6)	5 (41.7)
No	47/64 (73.4)	7 (58.3)
**Known TB contact**	(n=40)	(n=10)
Caregiver	16/40 (40.0)	4/10 (40.0)
Other household member	24/40 (60.0)	4/10 (40.0)
Other	0 (0.0)	2/10 (20.0)
**Known HIV exposure**		
Yes	20/70 (29.0)	2/13 (15.4)
No/unknown	50/70 (71.0)	10/13 (76.9)

A A small structure, often made of wood and placed in the backyard of a permanent structure;

B Data missing from one participant.

### Anticipated Stigma

Anticipated stigma was the most common stigma domain reported on the questionnaire, including fears of gossip (11/69; 15.9%) or their child’s feelings being hurt (11/70; 15.7%) (Figure). Caregivers also expressed concerns that others might undermine their children (10/70; 14.3%) or their children might lose friends (10/69; 14.5%) or respect in the community (7/70; 10%) if their TB diagnosis became known. Several caregivers recounted expecting negative social repercussions because of their child’s diagnosis during interviews. Some reported that because they had witnessed gossip about TB in their communities, they expected that their child, or potentially their entire household, would be stigmatized as well. One caregiver stated:

*“That is how it goes in the community; I have heard how people speak [negatively] about other people who have TB in the community, you know.”*

Another caregiver expected that people would distance themselves from her and her children because of the diagnosis:

*“[I thought] they would have said that their children might not play with my children, you know, or we can’t sit there by them.”*

Although no caregivers explicitly reported that this anticipation of stigma influenced their healthcare-seeking behavior, some described anticipated stigma influencing whether someone disclosed their TB diagnosis. A mother explained how she suspected a family friend who had TB did not initially disclose her diagnosis because *“she was wondering how the people will stare at her, or they might treat her differently.”*

**Figure.**
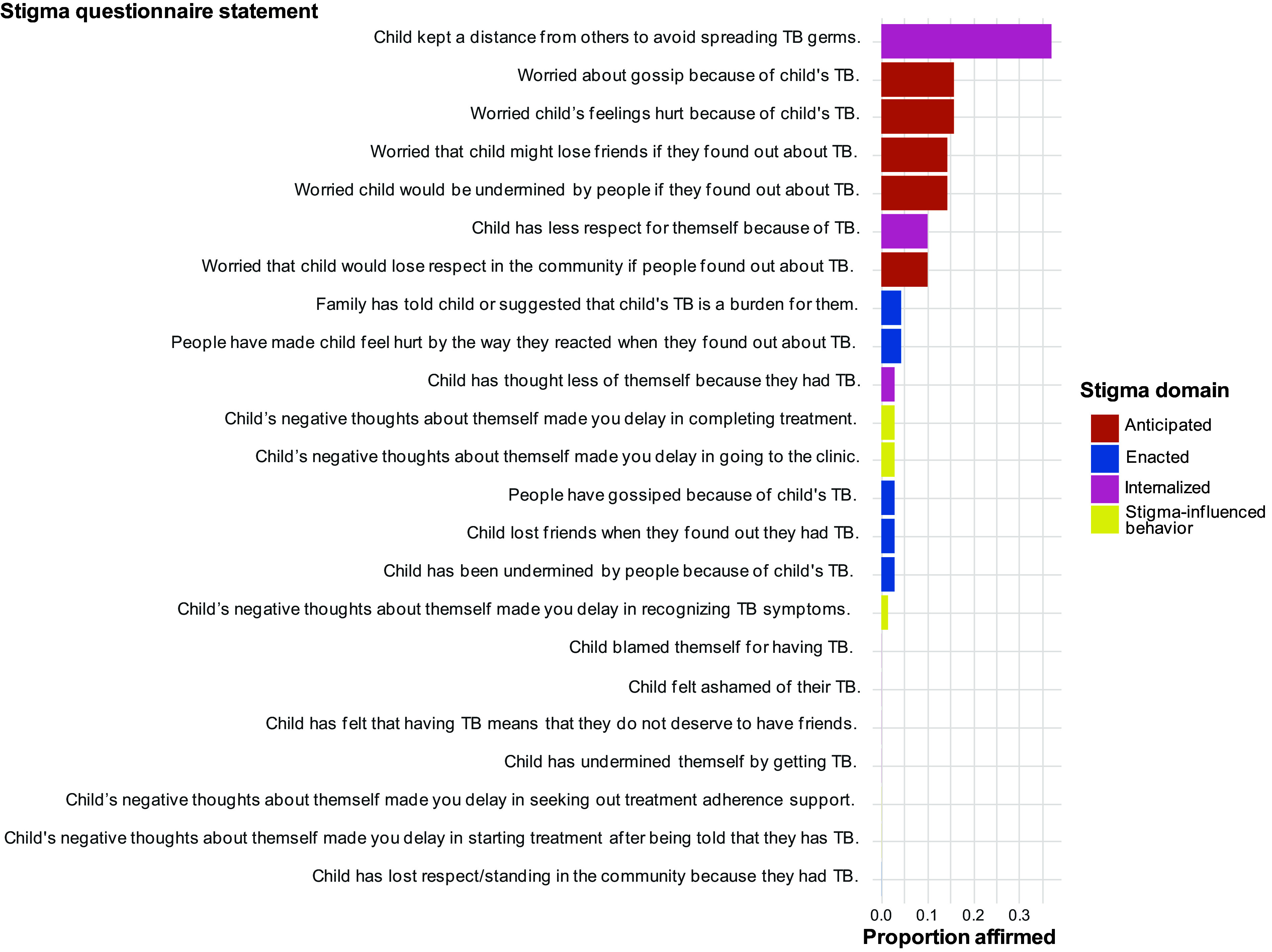
Proportion of affirmed stigma questionnaire statements (N=70 children).

### Enacted stigma

Relatively few responses to the questionnaire affirmed children experiencing enacted stigma. Affirmed experiences included family members suggesting the child's TB diagnosis was a burden (7/70; 4.3%), gossip due to TB diagnosis (2/70; 2.3%), the child being undermined by others due to TB diagnosis (1/70; 1.5%), and the loss of child’s friends (2/70; 2.3%) (Figure 1). No participants reported witnessing their children lose respect or standing in the community due to their TB diagnosis. Caregivers varied in their experiences of enacted stigma during interviews. Some caregivers affirmed that their child had experienced stigma, describing how their child was kept from other children while they had TB, often beyond the recommended quarantine for active TB illness. Some caregivers described TB stigma being enacted upon the entire household, even if members did not themselves have TB, particularly when it was assumed that an adult within the home had TB. One caregiver described the assumption that all household members had TB and the need to prove that she did not have TB:

*“…then I showed them I am negative cause I heard someone saying, everyone in this yard has TB.”*

Household-level stigma was also detailed when the entire household, including the caregiver, had TB. A father described how people’s dispositions to their entire family changed due to TB:

*“When we went to church, we would have to sit [to] one side.”*

Caregivers who denied that their children experienced stigma suggested that a child’s young age may have prevented them from experiencing stigma. A mother of a five-year-old suggested that, as an adult, she would have experienced TB stigma, while her son’s young age may have protected him from this isolation:

*“maybe there are people that would think like that … but most of the people … are alright with it because they don’t still worry because he’s still small/young … maybe I think if it was me … because I’m big/grown…they would’ve stayed away from me probably…”*

### Internalized stigma

Children keeping a distance from others to avoid spreading TB was most commonly affirmed across all domains (26/70; 37.1%); however, internalized stigma of the child, as perceived by their caregiver, was the least affirmed domain overall (Figure). Child losing respect for themselves because of their TB diagnosis was the second most affirmed internalized stigma experience (7/70; 10%). In contrast, no caregivers explicitly expressed that their child was exhibiting internalized stigma during interviews. Many suggested that young age was again protective against internalizing negative connotations associated with TB. One caregiver described how his 6 and 8-year-old sons would openly disclose their TB diagnosis seemingly without concern about others’ reactions:

*“I told the people I live with and my children, you know, the little ones don’t know what this is, so then they will scream, I have TB, and then people hear about it, you know, they weren’t allowed to play by some peoples’ houses…”*

Discussions of internalized stigma often shifted to caregivers’ feelings of shame because of their child’s illness. Several caregivers described experiences of internalized stigma themselves due to either their child’s TB diagnosis or their own. This often manifested in self-blame for the child’s illness and negative beliefs about themselves. One mother expressed doubting the quality of her parenting following her child’s TB diagnosis:

*“I started believing it, like every now and then but I tried to not let it bother me, in the beginning I blamed myself for my children getting TB and I sometimes believed that I was a bad mother.”*

Caregivers who had TB themselves often commented on the shame of giving TB to their children. One caregiver described how this led him to withdraw from his community and adopt negative beliefs about himself:

*“I started isolating myself and rejecting myself, you know, cause I had TB, and both my sons have to go through that, I lost my self-worth, I kept on thinking where did I get it, why did I get it, now I gave it to my sons.”*

### HIV stigma

Several interview participants brought up HIV stigma while discussing TB experiences, particularly when describing their own experiences with TB. One caregiver described the isolation he experienced when he contracted TB, comparing the isolation to HIV stigma:

*“*[People will] *treat you differently in the beginning, especially when you start losing weight, they remove you from the circle, they like put two and two together, it’s like you have AIDS or something.”*

## DISCUSSION

Caregiver responses provided valuable insights into the diverse manifestations and impact of anticipated, internalized, and enacted stigma. Anticipated stigma, manifested as a fear of mistreatment due to the TB diagnosis, was most commonly reported by caregivers of children with PTB and has frequently been observed in several other TB-stigma studies, including in South Africa.^15^ Caregivers did not commonly report internalized stigma in their children in either the survey or the interviews, indicating the challenge of assessing internalized stigma by proxy, rather than suggesting its absence among young children. More data are needed to explore whether young age is protective for stigma in a larger sample. Difficulty measuring young children’s perspective has been similarly noted in the HIV literature. Maternal mental health has been used as a proxy, particularly for perinatally transmitted cases.^26^ Caregivers frequently spoke of their own experiences, revealing feelings of self-blame, guilt, and isolation, which have been observed among caregivers of children in similar settings in South Africa, Zambia, and Ghana.^27,28^ The frequent shifts to caregiver stigma experiences during interviews, even among caregivers with slightly older, less dependent children, demonstrate the need for better measures to capture stigma experiences of young children and the impact of internalized stigma of caregivers on care-seeking for their children.

When caregivers described stigma enacted specifically upon their children, they often talked about more subtle forms, including prolonged isolation due to stigma. A study among adolescents with TB in Peru similarly observed isolation as a result of multiple TB-related forms of stigma.^29^ Often, caregivers described how the entire household experienced social ostracization and gossip due to their or their child’s TB diagnosis. This reinforces increasing recognition that stigma measures must consider multiple socio-ecological levels, including the individual, household, and community.^15^

The impact of TB stigma on health care-seeking behaviors was minimally described in this study; however, TB-related stigma is commonly known to influence engagement with TB prevention and treatment services.^15,30^ To better evaluate the impact of stigma among this population, future studies should consider levels of engagement with care and TB health outcomes, as well as possible sociodemographic factors that could influence the impact of stigma on the physical and psychosocial well-being of young children and their caregivers.

Strengths of this study include the novel discussion guide to elicit explicit conversations about TB stigma and the mixed-data methodology, which provides a richer overview of TB stigma. Quantitative analysis was limited due to the small sample size, preventing socio-demographic comparisons. In addition, recruitment from a clinical research setting may have influenced responses and participant knowledge of TB. Participant availability may have also influenced which caregivers were interviewed, although efforts were made to include all participants during the study window. In addition, although socio-behavioral scientists guided study counsellors, it is possible that social desirability may have biased participant responses, particularly about how stigma influenced care-seeking behaviors. Another limitation was the exclusion of children from participating in interviews due to the young median age of the sample. Although accounts of stigma were proxied, caregiver perspectives provided unique insights into their children’s experiences.

## CONCLUSION

This study provides a novel focus on the stigma experiences of young children with TB and their caregivers. Further investigation and development of validated integrated individual and household stigma measures is required to understand the impact of stigma on TB prevention and treatment, which will inform more effective, holistic interventions for TB and TB-related stigma.
